# Relation of pest insect-killing and soilborne pathogen-inhibition abilities to species diversification in environmental *Pseudomonas protegens*

**DOI:** 10.1038/s41396-023-01451-8

**Published:** 2023-06-13

**Authors:** Daniel Garrido-Sanz, Pilar Vesga, Clara M. Heiman, Aline Altenried, Christoph Keel, Jordan Vacheron

**Affiliations:** 1 Department of Fundamental Microbiology, University of Lausanne, CH-1015, Lausanne, Switzerland; 2 Present Address: Centro de Biotecnología y Genómica de Plantas (CBGP, UPM-INIA), Universidad Politécnica de Madrid (UPM)–Instituto Nacional de Investigación y Tecnología Agraria y Alimentaria (INIA), Madrid, Spain

**Keywords:** Microbial ecology, Comparative genomics, Functional genomics

## Abstract

Strains belonging to the *Pseudomonas protegens* phylogenomic subgroup have long been known for their beneficial association with plant roots, notably antagonising soilborne phytopathogens. Interestingly, they can also infect and kill pest insects, emphasising their interest as biocontrol agents. In the present study, we used all available *Pseudomonas* genomes to reassess the phylogeny of this subgroup. Clustering analysis revealed the presence of 12 distinct species, many of which were previously unknown. The differences between these species also extend to the phenotypic level. Most of the species were able to antagonise two soilborne phytopathogens, *Fusarium graminearum* and *Pythium ultimum*, and to kill the plant pest insect *Pieris brassicae* in feeding and systemic infection assays. However, four strains failed to do so, likely as a consequence of adaptation to particular niches. The absence of the insecticidal Fit toxin explained the non-pathogenic behaviour of the four strains towards *Pieris brassicae*. Further analyses of the Fit toxin genomic island evidence that the loss of this toxin is related to non-insecticidal niche specialisation. This work expands the knowledge on the growing *Pseudomonas protegens* subgroup and suggests that loss of phytopathogen inhibition and pest insect killing abilities in some of these bacteria may be linked to species diversification processes involving adaptation to particular niches. Our work sheds light on the important ecological consequences of gain and loss dynamics for functions involved in pathogenic host interactions of environmental bacteria.

## Introduction

Plant-associated pseudomonads are bacteria of great relevance in agriculture as they have the potential to protect crops from infections caused by plant pathogens and to promote plant growth through different mechanisms [1, 2]. Many plant-beneficial *Pseudomonas* belong to the *Pseudomonas fluorescens* species complex [3] that remains one of the largest and most diverse main lineages within the *Pseudomonas* genus [4, 5]. Indeed, it currently contains 134 identified species [6] out of the 302 validly published species within the *Pseudomonas* genus (https://lpsn.dsmz.de [7], accessed in October 2022), and it is further divided into 10 phylogenomic subgroups (SG) [3, 4, 8, 9]. One of these subgroups is the *Pseudomonas protegens* SG, which includes the type strain CHA0^T^, a model bacterium for the study of plant interactions, biocontrol and pest insect-killing mechanisms [1, 10–12]. In addition, this SG contains the three other named species *Pseudomonas saponiphila*, *Pseudomonas piscis* and the novel species “*Pseudomonas sessilinigenes*” [6, 13, 14], although the latter has not yet been validly published. Nonetheless, due to the increasing addition of novel genomes and the frequent lack of species assignation or misclassifications (e.g., *P. fluorescens*), the phylogenetic status of the *P. protegens* SG remains largely outdated, with studies encompassing only a few genomes [15]. Furthermore, these studies generally lack functional or phenotypic analysis to support differences at the genomic level. This calls for a revaluation of the *P. protegens* SG phylogeny, which together with comparative genome analyses can uncover a wider species diversity and differential distribution of relevant characters [6, 16], expanding the knowledge on the activities and ecological roles of members of the *P. protegens* SG.

Strains assigned to the *P. protegens* SG are known to colonise the rhizosphere of a wide range of plant species, including crops such as tobacco [12] or wheat [17], where they produce multiple secondary metabolites to antagonise soilborne phytopathogens. Among them, 2,4-diacetylphloroglucinol (DAPG) is a potent antimicrobial compound that also triggers inducible systemic defence responses of plants [12, 18]. Furthermore, two other typical antimicrobials produced by *P. protegens* strains, pyrrolnitrin and pyoluteorin, additionally contribute to their antagonistic effects towards phytopathogenic fungi and oomycetes [19–21]. *P. protegens* strains are not only able to colonise roots of plants, but other eukaryotic hosts, including insects and other arthropods where they are either commensal or pathogenic [22–25]. The insecticidal activity of *P. protegens* is multifactorial and involves a wide variety of mechanisms that allow the bacteria to establish in the gut, where they will compete with the resident gut microbiota causing microbiome dysbiosis [11], typically prior to invading the insect haemocoel. For an efficient systemic infection, bacteria also need to evade the immune responses of the insect, mediated by local production of reactive oxygen species and antimicrobial peptides in the gut, and immune cells once the gut epithelial barrier has been breached [26]. Once settled in the haemocoel, the bacteria cause a systemic infection by invading the haemolymph and producing the insecticidal Fit toxin and the antimitotic rhizoxin, which play crucial roles in the insect death [27, 28].

Although *P. protegens* strains have long been considered as bacteria associated with plants, they have also been isolated from other environments, for example, arthropods [23], nematodes [29, 30], freshwater fish [14] and occasionally the human respiratory tract [31]. This highlights their broad metabolic plasticity and their capacity to colonise multiple and diverse eukaryotic organisms [32]. Therefore, a phylogenomic study is necessary to better link the distribution of traits thus far associated to an environmental context and the ecology of these strains belonging to the *P. protegens* SG.

In the present study, we identified more than one hundred bacterial genomes belonging to the *P. protegens* SG distributed in 12 distinct species, many of which were previously unknown. Their species status was also supported by phenotypic traits, their ability to inhibit the growth of two soilborne phytopathogens, *Fusarium graminearum* and *Pythium ultimum*, and to kill the model pest insect *Pieris brassicae* (great white cabbage butterfly). We suggest that the presence or absence of these abilities is likely linked to species diversification processes and might be indicative of adaptation to particular niches.

## Materials and methods

### Identification of genomes belonging to the *P. protegens* phylogenomic subgroup

All *Pseudomonas* genome assemblies available from the NCBI RefSeq database were downloaded in April 2021. Genomes assigned to different lineages and distant species to *P. protegens*, (i.e., outside the *P. fluorescens* species complex [3]), were removed, resulting in a total of 2572 genomes. Intergenomic distances were calculated using the Genome-to-Genome blast distance phylogeny (GBDP [33]) online server, http://ggdc.dsmz.de) and using *P. protegens* CHA0^T^ as query. The obtained distances (Supplementary Table 1) showed a clear distinction between genomes from the *P. protegens* SG and the closely related *Pseudomonas chlororaphis* SG at a distance threshold of 0.135, which was used to keep 115 genomes.

### Phylogenomic analyses and genome clustering

Pairwise GBDP intergenomic distances of the 115 putative *P. protegens* SG genomes and other representative type strains of *Pseudomonas* genomes (184 genomes in total, listed in Supplementary Table 2) were transformed into a distance matrix and used to build a neighbour-joining phylogenomic tree, using FastME v2.1.6.1 [34]. *Escherichia coli* DSM 30083 ^T^ was used as outgroup. The tree was visualised and annotated using the interactive Tree Of Life (iTOL) v6 online tool [35]. Intergenomic distances were also used for a hierarchical clustering analysis at the species levels. The hclust R function and mclust v5.4.10 [36] and fpc v2.2-9 R packages were used to calculate single, average and complete-linkage clusters (*F* = 0, 0.5 and 1, respectively) using a range of distance thresholds (*T*) from 0 to the maximum distance and a step size of 0.0005. The number of species clusters was evaluated based on the highest Adapted Rand Index (ARI) score achieved across *F*s using a *T* = 0.036, which equals a digital DNA-DNA hybridization (dDDH) of 70% (Supplementary Table 3). The Type (Strain) Genome Server (TYGS; https://tygs.dsmz.de/) was used in September 2022 for a whole genome-based taxonomic analysis [37].

### Pangenome and functional characterization

OrthoFinder v2.2.6 [38], with DIAMOND v0.0.21.112 [39] searches and the MCL graph-clustering algorithm [40], was used on the amino acid sequences of the 115 genomes belonging to *P. protegens* SG. Single-copy amino acid sequences present in the 115 genomes were aligned with Clustal Omega v1.2.4 [41] and further concatenated. Blocks of poorly aligned columns and highly divergent regions were removed with gblocks v0.91b [42], using a minimum block of two amino acids. The results were imported into RAxML-NG v1.1.0 [43] for constructing a maximum-likelihood (ML) phylogeny, using the LG model of amino acid evolution with gamma-distributed substitution rates and the MRE-based bootstrap convergence criterion [44]. The tree was visualised and annotated with iTOL. The core-genome, pangenome and the genome-specific genomic fractions were calculated as previously described [16]. Faith’s phylogenetic diversity per species cluster was calculated using the ses.pd function of picante v.1.8.2 [45] R package, using 999 randomizations by shuffling taxa labels across tips of the GBDP phylogeny for the null community model.

The distribution of proteins or protein clusters of interest was examined by retrieving the orthologous groups (OGs) in which the amino acid sequence of *P. protegens* CHA0^T^ or any other representative genome was present. OGs belonging to non-specific wider groups were removed. The resulting number of OGs per cluster was used to calculate the percentage of genes present per genome. Results were represented using the ComplexHeatmap v2.9.2 R package [46].

A representative amino acid sequence from each OG, either belonging to *P. protegens* CHA0^T^ or the first one if CHA0^T^ was not present in the OG, was annotated using eggNOG-mapper v2.1.5 [47] against the eggNOG database v5.0.2 [48]. The last-rank KEGG BRITE hierarchical annotation was extracted using the KEGGREST v1.32.0 R package and relative abundances of BRITE categories per genome were calculated. Genomes with more than 85 contigs were discarded to avoid misrepresentation of categories. Bray-Curtis dissimilarities across strains were calculated using the vegdist function from the vegan v2.6-2 R package [49] and then a non-metric multidimensional scaling (NMDS) ordination analysis was performed with the metaMDS function, with a *k* = 3. Significance between groups was calculated using the pairwise permutational multivariate analysis of variance (PERMANOVA) of the adonis2 function included in the vegan R package and *p* values were adjusted using the false discovery rate (fdr) method.

### Bacterial strains, culture conditions and metabolic profiling

Twelve *Pseudomonas* strains, representative of eight species clusters identified in this study (Supplementary Table 4), were routinely cultured on nutrient agar and in nutrient yeast broth and incubated at 25 °C. The 12 strains were metabolically profiled using Biolog GEN III MicroPlate as described in full in the Supplementary information.

### Soilborne plant pathogen inhibition

The selected twelve *P. protegens* SG strains were tested for their antagonistic effect against the phytopathogenic fungus *Fusarium graminearum* Fg1 [50] and the oomycete *Pythium ultimum* Pu-11 [51]. The pathogen inhibition protocol is adapted from Besset-Manzoni et al. [52]. and is described in detail in the Supplementary information.

### Insect assays

To test the insect killing potential as well as the insect colonisation ability of the 12 representative *Pseudomonas* strains, we performed both oral and systemic infection assays on larvae of *Pieris brassicae*. Methods and data analysis are fully detailed in the Supplementary information.

### Analysis of the fit toxin genetic cluster

The *fit* gene cluster from *P. protegens* CHA0^T^ (*fitABCDEFGH*, from nucleotides 3,357,492 to 3,379,078, NCBI genome acc. no. NZ_LS999205.1) was used to retrieve the region from other strains of interest. Synteny was performed using cblaster v1.3.15 [53], with 70% amino acid identity over the full length of the CDS threshold and considering 70 kb upstream and downstream of the *fit* gene cluster. A ML tree of the *fit* cluster was constructed using the concatenated protein sequences as previously described. Pairwise blastn of the whole cluster was performed with blast+ v2.12.0 [54]. Results were used for clustering and to build heatmaps with the ComplexHeatmap R package.

## Results and discussion

### Phylogenomic analysis of the *P. protegens* subgroup reveals twelve distinct species clusters

**Fig. 1:**
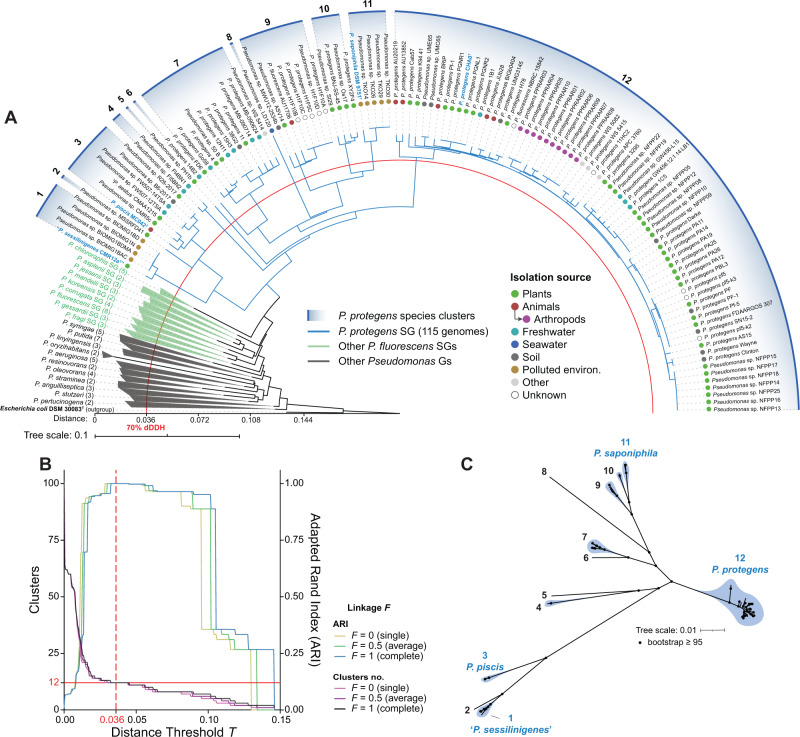
Phylogenomic analysis of the *Pseudomonas protegens* subgroup reveals twelve distinct species. **A** Neighbour-joining phylogeny of intergenomic distances of genomes belonging to the *P. protegens* phylogenomic subgroup (SG, in blue) and other representative type strain genomes of the remaining SGs from the *P. fluorescens* species complex (green) and other *Pseudomonas* phylogenomic groups (Gs, in grey). Numbers are shown according to species clusters identified in this study. Names in blue, bold and ^T^ denote type strains. Numbers in parentheses denote the number of genomes included in SGs or Gs. The red line indicates a distance of 0.036, which equals a digital DNA-DNA hybridization (dDDH) of 70%. Coloured dots represent the isolation source according to the NCBI BioProject description. **B** Hierarchical clustering analysis at the species level (dDDH ≥ 70%). The dashed red line denotes the maximum Adapted Rand Index (ARI) achieved across different distance thresholds (*T*) and linkages (*F*) analysed. The solid red line indicates the number of clusters identified at the maximum ARI. **C** Maximum-likelihood phylogenetic tree based on 2266 core single-copy amino acid sequences identified in the *P. protegens* SG. Numbers according to species clusters. The tree scale represents the number of substitutions per site. Black dots indicate a bootstrap ≥95%.

Amongst 2571 *Pseudomonas* genomes, we calculated intergenomic distances using the GBDP algorithm [33] and the genome of *P. protegens* CHA0^T^ as a query to first select those genomes that could belong to the *P. protegens* SG. Our results show that a distance threshold of 0.135 unambiguously separated genomes assigned to *P. protegens* SG from those of the closely related *P. chlororaphis* SG (Supplementary Table 1), resulting in 115 genomes putatively belonging to the *P. protegens* SG. Previous analyses using GBDP within 93 genomes of the *P. fluorescens* species complex determined a distance threshold of 0.1329 to separate the different *P. fluorescens* phylogenomic subgroups [3]. Genomes identified as putatively belonging to the *P. protegens* SG, were further used to calculate pairwise GBDP intergenomic distances to corroborate their phylogenomic subgroup adscription. The phylogenomic tree obtained (Fig. 1A) shows that the *P. protegens* SG constitutes a monophyletic group within the *P. fluorescens* species complex with its closest neighbouring subgroup being the *P. chlororaphis* SG. This phylogeny agrees with previous ones of the *P. fluorescens* species complex [3–5, 8] but substantially expands the number of genomes belonging to the *P. protegens* SG.

The clustering analysis of intergenomic distances (Fig. 1B; Supplementary Table 3) shows that at the species level (i.e., dDDH ≥ 70%, which equals a distance threshold *T* = 0.036), the *P. protegens* SG is composed of 12 species clusters. These 12 species are in total agreement (i.e., ARI = 1) with the reference partition using either single, average or complete linkage. The species status of the 12 clusters was validated using the TYGS (Supplementary Table 5), also showing the presence of subspecies in species clusters #7 and #12. The largest species cluster is *P. protegens sensu stricto* (i.e., referring to the species and not the SG), which is composed of 69 genomes including the type strain *P. protegens* CHA0^T^. Two other clusters contain named species: *P. saponiphila* (species cluster #11 [13]) and *P. piscis* (species cluster #3 [14]). The species cluster #1, was also previously identified and the name “*P. sessilinigenes*” was proposed using CMR12a^T^ as the type strain [6]. Additionally, there are four species clusters only represented by a single genome, which suggests that there may be more diversity to uncover. Moreover, through the *P. protegens* SG phylogeny, genomes have been incorrectly assigned to *P. protegens*, *P. fluorescens* species or unclassified isolates, which need to be renamed or formally named in the case of novel species. To substantiate the distinction within these 12 species clusters, we built a phylogeny based on 2,266 single-copy amino acid sequences present in all the genomes (Fig. 1C). The clustering pattern is identical to the one of the whole-genome phylogeny (Fig. 1A), identifying the same 12 species clusters (bootstrap support of ≥ 95%) and validating their status as separate phylogenomic clades (i.e., species clusters; species hereafter).

### The *P. protegens* SG pangenome and functional genome analysis evidence distinct species-specific features

**Fig. 2:**
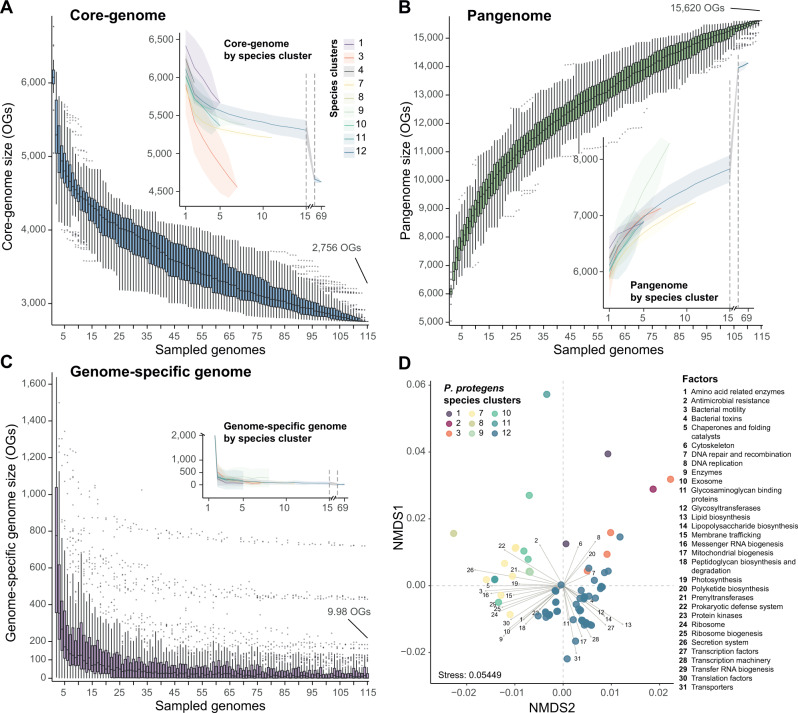
The *Pseudomonas protegens* SG pangenome and functional genome analysis evidence distinct species-specific features. Boxplots of the estimated sizes of the (**A**) core-genome, (**B**) pangenome and (**C**) genome-specific genome fractions represented as a function of the number of orthologous groups (OGs) identified over sequentially added genomes, using 100 replicates of randomly sampled genomes. Genome fractions per species cluster are included inside the panels: lines indicate mean values; shadows indicate standard deviations. Additional information can be found in Supplementary Table 6. **D** Non-metric multidimensional scaling (NMDS) of the relative abundances of BRITE KEGG functional categories annotated in *P. protegens* SG genomes. Genomes with more than 85 contigs were discarded. Arrows indicate fitted variables with a *p* value ≤ 0.05. Pairwise permutational multivariate analysis of variance (MANOVA) among *P. protegens* species clusters based on Bray-Curtis dissimilarities is listed in Supplementary Table 8.

The amino acid sequences of the 115 genomes belonging to the *P. protegens* SG were used to determine orthologous groups (OGs) and to calculate the core-genome, pangenome and genome-specific genomic fractions (Fig. 2A–C; Supplementary Table 6). The size of the *P. protegens* SG core-genome is smaller than the previous one calculated with only six genomes (2756 vs. 3631 OGs, [3]) but similar to other *P. fluorescens* SGs [16] and its cumulative curve shows a still-decreasing slope (Fig. 2A). The individual core-genome curves by species show a similar trend (Supplementary Table 6). However, the slope of the species #3 (*P. piscis*, seven genomes) is more pronounced, reaching a similar core-genome size than the one of the species #12 (*P. protegens sensu stricto*), composed of 69 genomes (4555 vs. 4625 OGs, respectively). This observation is not explained by the diversity of the species #3 and #12 (Faith’s PD of 0.03565 and 0.15499, respectively; Supplementary Table 7) and suggests that the discovery of more genomes from species #3 would have a great impact on the core-genome size of the *P. protegens* SG. The pangenome of the *P. protegens* SG is composed out of 15,620 OGs (Fig. 2B) and is still “open”: it will continue to increase, as newly discovered genomes add on average about 10 new genes to the pangenome pool (i.e., genome-specific genome fraction; Fig. 2C). A previous study of the *P. corrugata* SG (also belonging to the *P. fluorescens* species complex) achieved a similar pangenome size, consisting of 16,530 OGs [16], which also agrees with the ones reported in other *Pseudomonas* lineages, such as *P. syringae* (pangenome of 12,829 OGs using 19 strains [55]). Furthermore, similar tendencies are observed in the individual curves by *P. protegens* SG species, although the pangenome of species #9 (eight genomes) is comparably the highest, containing *ca*. 1,000 OGs more than the remaining species at the same number of sampled genomes (Fig. 2B). Indeed, genome-specific curves (i.e., new OGs that appear over sequentially added genomes) also show the highest number in species #9 (average of *ca*. 276 with eight genomes) compared to the rest of the curves at the same number of sampled genomes, indicating a higher intraspecific genetic diversity within this species compared to others. Open pangenomes of steadily increasing OGs result from high genetic exchange which is usually found in bacteria with the ability to colonise multiple environments, which is in support of the metabolic versatility of *Pseudomonas* species [56], and reflects the ecology of soil-dwelling bacteria [57].

To explore if the differences between the *P. protegens* SG species also extend to the functional level, we performed a NMDS based on the relative abundances of BRITE functional categories annotated in the studied genomes. The ordination results (Fig. 2D) and the statistical analysis (Supplementary Table 8) demonstrate that species #12 (*P. protegens sensu stricto*) is functionally different from the rest of the *P. protegens* SG species (adj. *p* value ≤ 0.05) with differences in lipid and lipopolysaccharide biosynthesis, as previously observed [58, 59], bacterial toxins or transcription factors, among other functions (Fig. 2D). The differences between other groups were not as distinctive. However, species #3 and #12 are separated from species #7 to #11 with main differences in functions related to secretion systems, prokaryotic defence systems or motility.

**Fig. 3:**
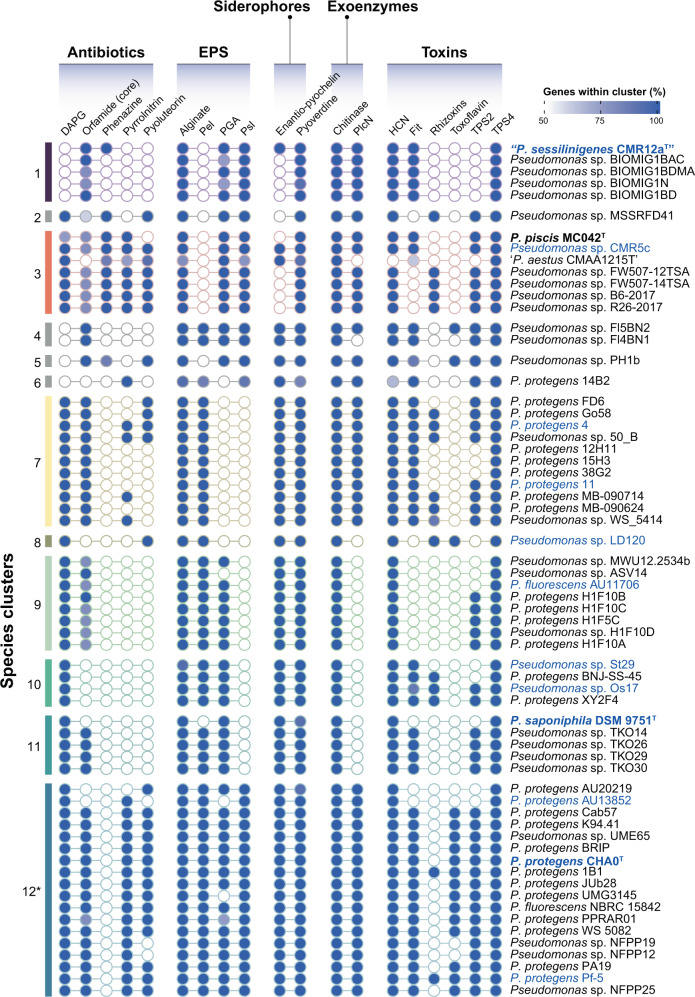
Distribution of characters of interest in *Pseudomonas protegens* SG genomes. The scale in blue shows the percentage of proteins within the protein cluster present in the strain. Only percentages ≥50% are considered as the presence of the character. Bold and ^T^ indicate type strain. The strains phenotypically characterised in this study are highlighted in blue. *Only 20 representative genomes of species #12 are shown. For detailed information, including specific proteins used for character description and their distribution in the 115 *P. protegens* SG genomes analysed in this study, refer to Supplementary Fig. 1.

We also analysed the distribution of characters relevant for antagonism with soilborne phytopathogens and insect pathogenicity within the *P. protegens* SG (Fig. 3; detailed information can be seen in Supplementary Fig. 1). Common traits previously reported in strains belonging to the *P. fluorescens* species complex [3, 23, 60, 61] are also encoded in most genomes of the *P. protegens* SG, including the production of the biocide hydrogen cyanide, the siderophore pyoverdine and the exopolysaccharide alginate. We have identified that certain characters previously presumed to be common to all *P. protegens* SG genomes, follow in fact a species-specific distribution. For example, out of the 12 species identified in this study, four do not possess the biosynthetic cluster for the antimicrobial compound DAPG. Indeed, strains belonging to the “*P. sessilinigenes*” species (cluster #1), the species #4, #5 and #6 lack this operon (Fig. 3). This polyphyletic distribution has been previously reported in DAPG-producing pseudomonads [62]. Other antimicrobials such as pyrrolnitrin and pyoluteorin [19, 20] are absent from species #1, #4, #9 and #10 and partially absent in species #7. Phenazines are broad-spectrum antimicrobial compounds widely characterised in *P. chlororaphis* [63], that we only found in all strains from species #3, the single-species #2 and #5 and only in strain CMR12a^T^ from species #1. Orfamide-type cyclic lipopeptides (CLP) are surface-active metabolites with broad activity spectrum characterised in *Pseudomonas* spp [64]. that have been shown to also target insects [65]. In our study, the orfamide biosynthetic gene cluster had a widespread distribution, except for species clusters #6, #8 (strain LD120), #10 and certain strains from the species clusters #3 and #12 (including AU13852). Furthermore, although the exopolysaccharides loci Pel (pellicle locus), PGA (poly-N-acetyl-glucosamine) and Psl (polysaccharide synthesis locus) were previously reported to be present in all *P. protegens* genomes examined [61, 66], our results show that only species #12 (*P. protegens sensu stricto*) harbours all the three exopolysaccharides loci, while the distribution in the remaining species is scattered (Fig. 3). Finally, another trait that has a strong polyphyletic distribution is the insecticidal toxin Fit [28], which is absent from species #2, #8, #9 and other specific strains from the remaining groups. The phylogenetic distribution of these traits could be related to the ability of these species or individual strains to exploit particular niches [67].

### Variable insecticidal activity by *Pseudomonas protegens* strains suggests niche specialisation

*P. protegens* SG strains also protect plants by killing pest insects or impacting their development [15, 25]. Oral intake of bacteria by pest insects allows them to establish within the insect intestinal tract [24, 25, 29, 68]. From there, *P. protegens* CHA0^T^ has been shown to cross the gut epithelial barrier to reach the insect circulatory cavity (haemocoel), where it proliferates and produces the Fit toxin causing septicaemia and ultimately killing the insect [11, 22, 25]. To understand whether this ability might be linked to species-specific characters within the *P. protegens* SG, we selected 12 representative strains that were available in our collection or shared by other research groups. These strains cover 8 of the 12 species described above and are listed in the Supplementary Table 4. We tested the ability of these *P. protegens* SG strains to kill *P. brassicae* larvae, and to colonise the gut and the haemolymph of the larvae upon oral administration and haemocoel injection assays (Supplementary Table 9 and 10).

**Fig. 4:**
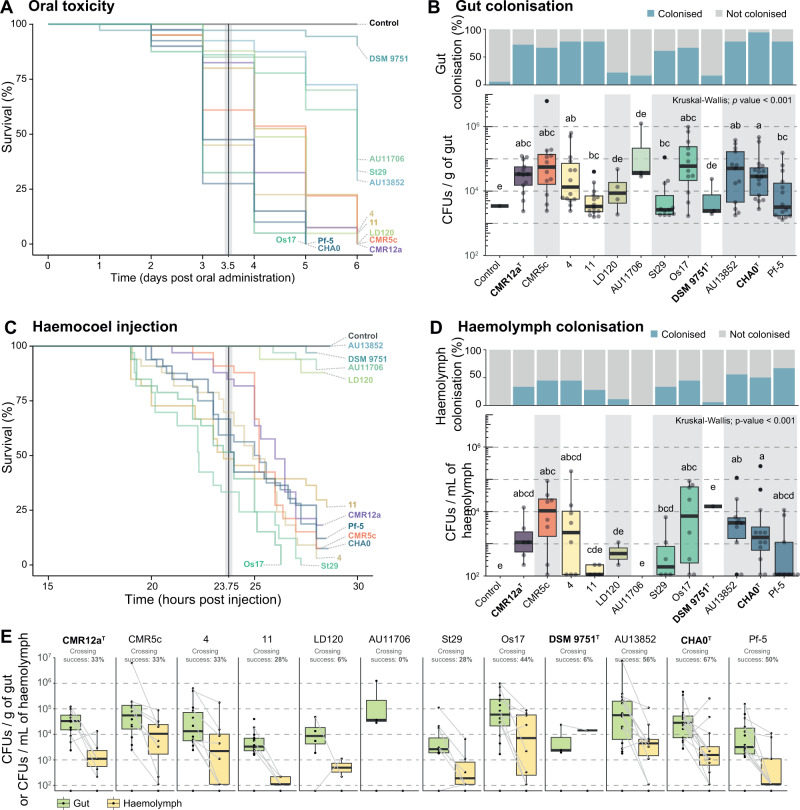
Insecticidal activity of representative *Pseudomonas protegens* SG strains towards larvae of the pest insect *Pieris brassicae*. **A**, **B** Kaplan-Meier survival plots of larvae of *P. brassicae* when exposed to the twelve *P. protegens* SG strains tested, either by oral administration or by haemocoel injection. The plots represent the likelihood that the insects survive through time following the administration of the different *P. protegens* strains. Statistical differences of the survival curves (Log-rank test; *p* value < 0.05) are shown in Supplementary Table 9. **C**, **D** Colonisation of the pest insect guts (**C**) or haemolymph (**D**) 24 h post feeding. The barplots represent the number of larvae colonised (blue) or not colonised (grey). The boxplots below show the number of CFUs per g of gut or per mL of haemolymph recovered. Statistically significant groups (*p* value < 0.05) are indicated with different letters based on Kruskal-Wallis with post hoc testing. **E** Paired boxplots and gut-haemolymph crossing success per strain. Strain names highlighted in bold correspond to type strains.

Under oral administration, we observed a high variability in the larval mortality (Fig. 4A, Supplementary Fig. 2). However, three different killing behaviours could be distinguished. The strain DSM 9751 ^T^ was unable to kill the insects, while strains AU11706, AU13853 and St29 displayed an intermediate killing activity. All other strains were able to kill the insects by six days post oral administration. These results can be explained by the ability of the strains to first establish within the insect gut and then cross the gut epithelial barrier into the haemocoel (Fig. 4). The strains DSM 9751 ^T^ and AU11706 were unable to colonise the gut of the larvae (Fig. 4B) and consequently they could not reach the haemocoel (Fig. 4D, E). Even directly injected into the haemolymph, both strains could not kill the insects (Fig. 4C). Conversely, strain AU13852 efficiently established within the gut of the insect and colonised the haemocoel with a 56% success rate (Fig. 4B, D, E). However, it was unable to kill the insects when directly injected into the haemocoel, and a delayed mortality was observed when it was administered orally (Fig. 4A, C). Finally, strain LD120 was able to kill the insects following oral intake but it showed a lower colonisation capacity in both the gut and the haemocoel (Fig. 4B, D). The inability of these four strains (DSM 9751 ^T^, AU11706, AU13852 and LD120) to kill the insects following haemocoel injection is likely associated with the absence of the *fit* operon (Fig. 3), as well as their inability to evade the immune system of the insects. Indeed, all the strains were able to grow in Grace’s insect medium, mimicking the haemolymph composition (i.e., without the influence of the immune system of the insect, Supplementary Fig. 3). However, the reason why strain LD120 was able to kill the insects only upon oral administration remains to be elucidated. One explanation could be that strain LD120 disturbs the insect gut microbiome, producing a dysbiotic state that ultimately kills the insects. Nonetheless, these four strains might potentially have lost the ability to kill insects as a consequence of adaptation to particular niches. Indeed, their isolation sources are unrelated to plants or insects (Fig. 1; Supplementary Table 4). The strains AU13852 and AU11706 were isolated from the human respiratory tract of patients suffering from cystic fibrosis [31], while strain LD120 was isolated from the blade of a marine brown alga [67]. Finally, strains that harbour the Fit toxin gene cluster (CMR12a^T^, CMR5c, 4, 11. St29, Os17, CHA0^T^ and Pf-5) were all able to cross the gut epithelial barrier and kill the insect (Fig. 4). Furthermore, comparisons between strains belonging to the same species clusters showed differences under injection and/or oral intake regarding the final larval survival (strains 11 and 4 in injection, strains St29 and Os17 in oral intake or strain AU13852 in injection and oral intake compared to strains CHA0^T^ and Pf-5, Fig. 4A, C). The absence of rhizoxin, a potent antimitotic [69], from strains 11, St29 and AU13852 (Fig. 3) could also explain these differences, but not in the case of strains CHA0^T^ and Pf-5, exposing the multifactorial nature of the insecticidal activity in *Pseudomonas* [70].

The insect killing ability was previously thought to be particular to specific *Pseudomonas* phylogenetic subgroups [15, 71]. However, our study demonstrates that within subgroups and even within a given bacterial species cluster, contrasted insecticidal activity can be detected, which could be related to their isolation origin. Indeed, the strains that were isolated from clinical samples did not demonstrate insecticidal activity although they are part of a species cluster previously described as insecticidal [23], which includes other members displaying this behaviour (e.g., strains CHA0^T^, Pf-5). Therefore, it is likely that the adaptation to a new host (human, algae) leads to a loss of functions such as the ability to colonise the insect and/or produce the Fit toxin that kills insects.

### The conservation of the *fit* toxin operon follows species diversification events

**Fig. 5:**
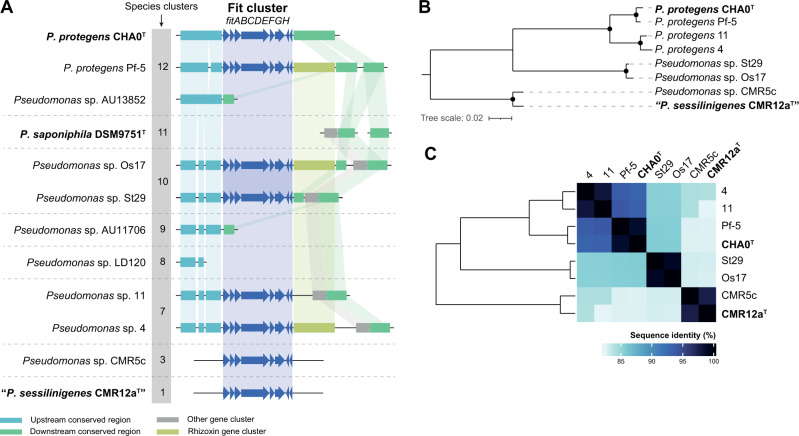
Analyses of the Fit toxin gene cluster in representative *Pseudomonas protegens* SG genomes. **A** Synteny of the Fit toxin gene cluster (dark blue, at scale) and genetic context (not at scale) in the 12 *P. protegens* SG genomes phenotypically characterised. Conserved regions are represented as coloured rectangles. For detailed information, refer to the Supplementary Fig. 4. **B** Phylogenetic ML tree of the concatenated FitABCDEFGH amino acid sequences. Black dots represent bootstrap values equal to 100%. The scale represents the number of substitutions per site. **C** Heatmap of the entire *fit* gene cluster (nucleotide) representing sequence identity values obtained by blastn.

The Fit toxin has been previously identified as an important insecticidal toxin in plant-associated *Pseudomonas* against several species of Lepidoptera [28, 68, 72]. The toxin is not expressed in the gut of the insect [61], while it is expressed in the haemolymph suggesting a role in systemic infections [73, 74]. All strains tested that were able to kill *P. brassicae* larvae in injection assays harbour in their genome the *fit* gene cluster (*fitABCDEFGH*), while closely related or distant strains that were unable to kill the insect upon injection, lack these genes (Fig. 3), confirming a major role of this cluster in insect pathogenicity [28, 68]. To further identify differences at the genetic level of the *fit* gene cluster that could point to species-specific differences in the insect killing abilities of the tested strains, we analysed the synteny of this cluster. The results show a high degree of conservation of the *fit* gene cluster (≥82.25% of nucleotide sequence identity) as well as conservation in the flanking regions in most of the strains (Fig. 5, Supplementary Fig. 4), except in strains CMR5c and CMR12a^T^. Moreover, in some strains, the downstream region is characterised by the presence of a rhizoxin gene cluster. Rhizoxins are potent eukaryotic antimitotic compounds previously identified in *P. protegens* Pf-5 [69, 75]. The region comprising both the Fit toxin and the rhizoxin gene clusters has been previously identified as a genomic island [71]. The absence of the rhizoxin gene cluster in closely related strains within the same species (species #3, #7, #10 and #12; Fig. 3; Supplementary Fig. 1) highlights the high strain-specific diversity of *P. protegens* SG genomes [76] and could explain differences in their insect killing behaviour (Fig. 4). This also suggests reorganisation events affecting this island after its acquisition. Furthermore, the fact that both CMR5c and CMR12a^T^ strains do not share the same genetic context compared to the remaining strains (Fig. 5A) supports the hypothesis of at least two independent acquisition events. The acquisition or loss of genes is a powerful driver of bacterial diversification [77, 78]. The importance of this island in killing insect hosts make it a good candidate marker for screening new isolates to be used as pest control agents.

### Antagonistic activity against soilborne pathogens is not linked to specific patterns of antimicrobial functions

**Fig. 6:**
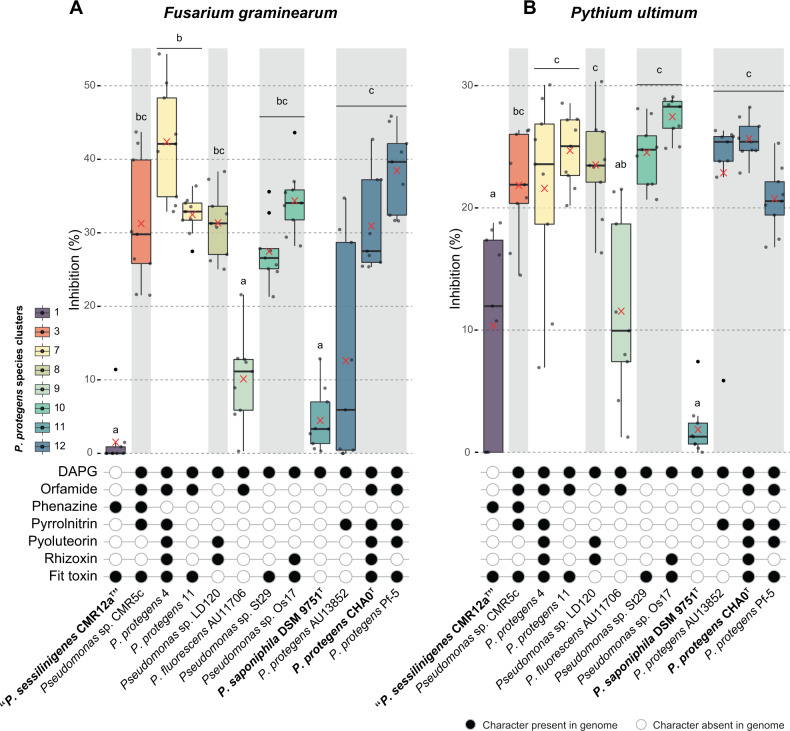
Inhibition of soilborne phytopathogens by *Pseudomonas protegens* SG strains. Boxplot showing the percentage of inhibition of (**A**) *Fusarium graminearum* growing on potato dextrose agar (PDA) plates and (**B**) *Pythium ultimum* growing on malt agar (MA) plates calculated by comparing the phytopathogenic fungus or oomycete growth area in presence of the bacterial strain versus the control without bacteria. Colours are according to species clusters identified in this study. Mean values are indicated with a red cross. Statistical differences between species clusters were calculated using the Kruskal-Wallis rank sum test with Dunn’s post hoc correction. Differences between groups are shown with different letters (*p* value < 0.05; for additional information and detailed strain comparisons refer to Supplementary Table 11). The distribution of main antifungal characters detected in the genome of the 12 strains are reported below the boxplots. Strain names highlighted in bold correspond to type strains.

One of the major plant-beneficial traits attributed to *P. protegens* SG strains is their ability to antagonise soilborne phytopathogens [1, 12, 25, 79]. We assessed the ability of these strains to inhibit the growth of two typical soilborne phytopathogens, the fungus *F. graminearum* and the oomycete *P. ultimum*. Most of the strains were able to inhibit the growth of both plant pathogens, except the strains CMR12a^T^, AU11706 and DSM 9751 ^T^, which might be due to the lack of DAPG (in strain CMR12a^T^), pyrrolnitrin and pyoluteorin biosynthetic operons in these three strains (Fig. 6; average inhibition of 27.5% to 42.3% against *F. graminearum* and 20.6% to 27.4% against *P. ultimum*). Despite this, the strain CMR12a^T^ was previously shown to inhibit *Pythium myriotylum* root rot disease in cocoyam (*Colocasia esculenta*) rhizosphere, from where the strain was isolated [79], suggesting that other genetic determinants are involved in its specialisation towards this specific pathogen. When comparing strains from the same species cluster, no statistical differences were found between them (Supplementary Table 11). Therefore, pathogen inhibition may not only be dependent on the presence or absence of one specific or a group of antimicrobial compounds but also on additional factors that could be strain specific. In this line, the level of expression of these antimicrobials as well as their specific combinations within genomes might intervene in the observed phenotypes. All the *Pseudomonas* tested were able to grow on the two media used for the inhibition assays, and growth rates under different pHs remained similar (Supplementary Fig. 5). Thus, growth differences are unlikely to be a confounding factor in explaining the inhibition abilities against *F. graminearum* and *P. ultimum*. Nonetheless, our results demonstrate that most of the selected strains from the *P. protegens* SG were able to antagonise soilborne phytopathogens, making them interesting as potential biocontrol agents. However, whether this antagonism towards the two pathogens is common to all the strains of these species or if it is rather particular to the strains analysed in this work remains to be studied.

### *P. protegens* SG species have different overall metabolic and antibiotic resistance profiles

**Fig. 7:**
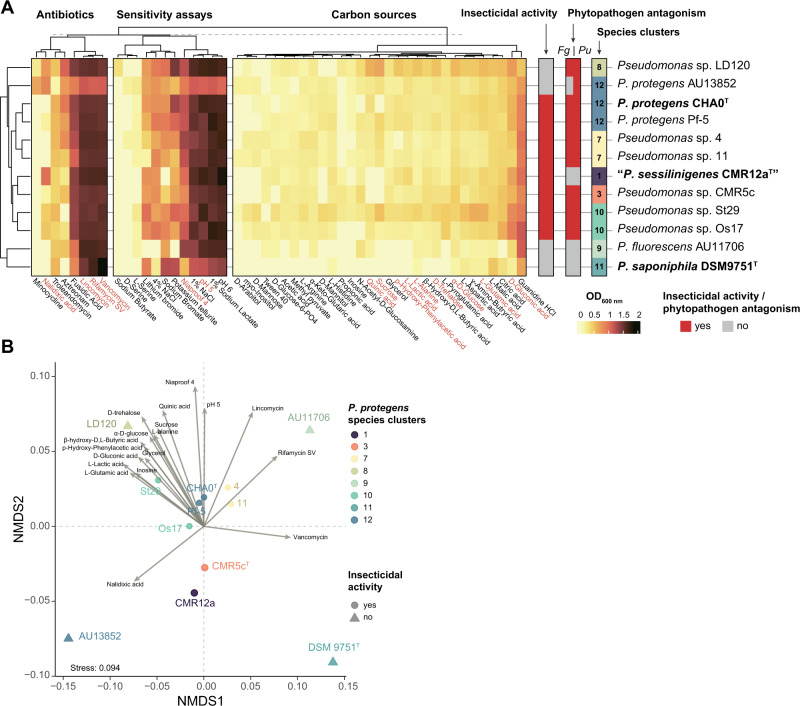
Phenotypic differences between the twelve representative *Pseudomonas protegens* SG strains. **A** Heatmap and clustering analyses of the Biolog results. The Biolog data used to generate these results correspond to the carrying capacity (i.e., the maximum OD_600_ observed during the growth kinetic). Columns were split according to the three main phenotypic categories, i.e., antibiotic resistance, sensitivity assays and carbon sources usage. Categories written in red indicate those with a *p* value ≤ 0.05. **B** NMDS of Biolog GEN III performed in 12 representative genomes belonging to eight *P. protegens* SG species clusters and measured as growth expressed as OD_600_ nm values. Fitted variables with a *p* value ≤ 0.05 are represented as arrows. Insecticidal activity of the strains towards *P. brassicae* larvae in injection assays is indicated as circles (strain able to kill the insect) or triangles (strain uncapable of killing the insect). *Fg*: *Fusarium graminearum*, *Pu*: *Pythium ultimum*.

We also phenotypically characterised the 12 strains representative of eight *P. protegens* SG species clusters (Supplementary Table 4) by using Biolog GEN III Microplate. The clustering pattern (Fig. 7A) was similar to the one observed in genome-based phylogenies (Fig. 1A, C). The only strain that was separated from its species cluster is *P. protegens* AU13852, mainly due to differences in antibiotic resistance and sensitivity assays, which suggest subspecific diversification, as also observed in the TYGS analysis (strain AU13852 belongs to a different subspecies than other strains from *P. protegens sensu stricto*; Supplementary Table 5). This separation of strain AU13852 at the functional level might be attributed to its ability to colonise the human respiratory tract [31] and its lack of insecticidal activity (Fig. 4). Biolog data were also used for an NMDS ordination analysis. Although strains clustered together by species adscription (Fig. 7B), four strains were different: LD120, AU11706, AU13852 and DSM 9751 ^T^. The ordination pattern of these strains is mainly supported by their ability to resist to specific antibiotics (i.e., nalidixic acid, vancomycin, rifamycin SV and lincomycin) and the differential utilisation of carbon sources. One explanation in the case of strains AU11706 and AU13852 could be attributed to their isolation source from cystic fibrosis patients [31], often treated with different antibiotics for secondary bacterial infections or as a prophylactic treatment [80]. Strain LD120 is the only known *Pseudomonas* strain ever isolated from the marine brown algae *Saccharina latissima* (Kingdom *Chromista*) [67]. The colonisation of this host could imply profound metabolic changes, as observed in its metabolic profile (Fig. 7A) and a divergent evolutionary lineage (Fig. 1A, C).

## Conclusion

In this work, we have shown the presence of 12 species within the *Pseudomonas protegens* SG based on comparative genome analyses. Among these, only four have been previously described: *P. protegens sensu stricto*, *P. saponiphila*, *P. piscis* and “*P. sessilinigenes*”. The species identified in this study substantially increase the known diversity of this bacterial group. The differences between these species also extend to the functional level, both in the distribution of annotated features and in phenotypic characteristics. Although most strains were also able to kill the plant pest insect *P. brassicae*, four strains failed to do so. These strains were isolated from uncommon *P. protegens* niches: *P. protegens* AU13852 and *P. fluorescens* AU11706 from humans, *Pseudomonas* sp. LD120 from a seawater alga, while the origin of *P. saponiphila* DSM 9751 ^T^ remains unknown, though its description suggests a niche related to polluted soil or groundwater. The lack of the Fit toxin gene cluster in these four strains and/or their inability to breach the gut epithelial barrier and establish in the haemolymph explain their non-insecticidal behaviour, which could point to potential specific niche adaptations that could either affect their entire species cluster (strain AU11706) or adaptations at the subspecies level (strains AU13852 and DSM 9751 ^T^).

The results presented in this work expand the knowledge on the ecological roles that known and novel *P. protegens* SG species have in relation with two hosts, plants and insects. The multiplicity of hosts that *P. protegens* SG strains can colonise or protect is reflected on an open and diverse pangenome, which will likely grow with the addition of novel genomes. Its consequences point to specialised niche exploitation, either antagonising soilborne plant-pathogens or by producing systemic pest insect infections, thus protecting the plant host. Conversely to this presumed common phenotype to *P. protegens* SG strains, the lack of phytopathogen antagonism and insecticidal behaviour in some strains shows potential specialised evolutionary paths to cope with other niches (e.g., aquatic organisms or humans). This knowledge on *P. protegens* SG might guarantee a more efficient selection of strains of this group to be used as biocontrol agents in agriculture and opens the possibility of finding novel niches for *P. protegens* SG strains. Altogether, our analysis reveals a dynamic interplay between the acquisition and loss of traits, as well as the specific variation of these traits among closely related strains as a driver of species diversification. These findings underscore the delicate balance between adaption, niche differentiation, and the emergence of new ecological strategies.

## Supplementary information

Supplementary information

Supplementary tables

## Data Availability

The data used for this study is publicly available and references and accession numbers are provided throughout the text or in the supplementary material when necessary.
