# Supplementary material for: Relation of pest insect-killing and soilborne pathogen-inhibition abilities to species diversification in environmental Pseudomonas protegens
Source: ISME J. 2023 Jun 13;17(9):1369–81. doi: 10.1038/s41396-023-01451-8 (PMC10432460; doi:10.1038/s41396-023-01451-8)
Supplement: Supplementary file 1 — Supplementary information [file 41396_2023_1451_MOESM1_ESM.docx]

**SUPPLEMENTARY INFORMATION**

**Relation of pest insect-killing and soilborne pathogen-inhibition abilities to species diversification in environmental *Pseudomonas protegens***

Daniel Garrido-Sanz^1#^, Pilar Vesga^1†#^, Clara M. Heiman^1^, Aline Altenried^1^, Christoph Keel^1#^ and Jordan Vacheron^1#^

^1^ Department of Fundamental Microbiology, University of Lausanne, CH-1015 Lausanne, Switzerland

^†^ **Current address:**

Centro de Biotecnología y Genómica de Plantas (CBGP, UPM-INIA), Universidad Politécnica de Madrid (UPM)–Instituto Nacional de Investigación y Tecnología Agraria y Alimentaria (INIA), Madrid, Spain.

**^#^ Corresponding authors:** Daniel Garrido-Sanz ([daniel.garridosanz@unil.ch](mailto:daniel.garridosanz@unil.ch)), Pilar Vesga ([pilar.vesga@upm.es](mailto:pilar.vesga@upm.es)), Christoph Keel ([christoph.keel@unil.ch](mailto:christoph.keel@unil.ch)) and Jordan Vacheron ([jordan.vacheron@unil.ch](mailto:jordan.vacheron@unil.ch))

**Supplementary Methods**

**Bacterial strains, culture conditions and metabolic profiling**

**Soilborne plant pathogen inhibition assays**

**Bacterial growth assessment in Grace’s insect medium**

**Insect assays**

**References**

**Supplementary Figures**

**Supplementary Figure 1**. Distribution of traits of interest in 115 *Pseudomonas protegens* SG genomes.

**Supplementary Figure 2**. Mortality of *Pieris brassicae* larvae upon feeding or injection with 12 *Pseudomonas* strains.

**Supplementary Figure 3**. Growth parameters of the 12 *Pseudomonas* strains in Grace’s insect medium.

**Supplementary Figure 4**. Syntenic organisation of the Fit toxin gene cluster and flanking regions in the 12 *Pseudomonas* strains analysed.

**Supplementary Figure 5.** Growth parameters of the 12 selected *Pseudomonas* strains at different pH based on data extracted from the Biolog plates.

**Supplementary Methods**

**Bacterial strains, culture conditions and metabolic profiling**

Twelve *Pseudomonas* strains, representative of eight species clusters identified in this study (listed in **Supplementary Table 4**, were routinely cultured on nutrient agar (NA) and in nutrient yeast broth (NYB) and incubated at 25 °C. The 12 strains were metabolically profiled using Biolog GEN III MicroPlate. Each well of the 96-well plates was part-filled with 95 µL of minimal medium (MM) without carbon source (for 1 L; 1 g of NH_4_CL; 3.49 g of Na_2_HPO_4_·2H_2_O; 2.77 g of KH_2_PO_4_). The optical density at 600 nm (OD_600_) was measured each two minutes for 10 min using a Synergy H1 plate reader (BioTek, USA) to obtain blank values. Then, each well was inoculated with 5 µL of a bacterial suspension adjusted in MM to an optical density at 600 nm (OD_600_) of 1 (i.e., final OD_600_ of 0.05) and the growth kinetic was measured every 5 min for 24 h. The bacterial suspensions were obtained from an overnight NYB culture, restarted (1:100 v/v) in fresh medium until they reached an OD_600_ of 0.5 - 0.6. Then, cells were pelleted and washed with MM before inoculation into the wells. Biolog data was processed prior to analysis by subtracting the blank values for each well, as well as the negative growth curve control. Compounds for which all strains reported a growth below a minimum threshold of OD_600_ of 0.1 were filtered out. A heatmap and a clustering analysis were then performed with the ComplexHeatmap R package, using Euclidean distances and splitting rows by main Biolog categories. In addition, a NMDS ordination analysis was performed using Bray-Curtis dissimilarities as described in the main text using a *k* = 2.

**Soilborne plant pathogen inhibition assays**

The phytopathogenic fungus *Fusarium graminearum* Fg1 [1] and the oomycete *Pythium ultimum* Pu-11 [2] were grown and maintained on potato dextrose agar (PDA) or malt-agar (MA) plates, respectively, at room temperature. To test the antifungal activity of the selected *Pseudomonas* strains, we used a protocol adapted from Besset-Manzoni et al. [3]. Briefly, an 8 mm mycelial plug of each pathogen was placed at the centre of a PDA or MA plate. Overnight cultures (25 °C and 180 rpm) of the bacterial strains were washed in 0.9% NaCl solution and the resulting bacterial suspensions adjusted to OD_600_ of 0.1. One µL of bacterial suspension (~10^5^ bacterial cells) was streaked at 2 cm from the mycelium and along a line of 3 cm. Controls were done by streaking 1 µL of sterile 0.9% NaCl solution. All the plates were incubated at room temperature. The experiment ended when each pathogen entirely colonised the control plate without bacteria. Pictures were taken to quantify pathogen growth. The growth area of the pathogens was measured with ImageJ software v1.53t. Three independent experiments with three technical replicates per pathogen and bacterial strain were performed. Differences in pathogen growth when exposed to the different bacterial strains were assessed using the non-parametric test of Kruskal-Wallis rank sum (stats v.4.2.1) and the post hoc Dunn’s test with the Holm correction for the adjusted *p*value (FSA v.0.9.3). Significant differences were considered when *p*value < 0.05.

**Bacterial growth assessment in Grace’s insect medium**

The growth of the 12 selected *Pseudomonas* strains was assessed in Grace’s Insect Medium (GIM, Sigma-Aldrich), a medium mimicking the composition of insect haemolymph. Overnight cultures of the 12 *Pseudomonas* strains growing in NYB were restarted (1:100 v/v) in fresh NYB until they reached an OD_600_ of 0.5 - 0.6. Bacterial cells were pelleted and washed with 0,9% NaCl solution and adjusted to an OD_600_ of 2. Five µL of each *Pseudomonas* strain suspension were inoculated into 195 µL of GIM (Final OD_600_ of 0.05) contained per well in a 96-well plate. The bacterial growth was measured every 15 min for 24 h using a Synergy H1 plate reader (BioTek, USA). The growth parameters were calculated using the GrowthCurver R package [4].

**Insect assays**

*Pieris brassicae* larvae were reared on pesticide-free cabbage (*Brassica oleracea*) at 60% of relative humidity, 25 °C, 16 / 8 h light / dark photoperiod until they reached the desired instar.

*Feeding*

Twelve third-instar *Pieris brassicae* larvae were used to assess oral insecticidal activity of the 12 selected *Pseudomonas* strains following an adapted protocol from Flury and colleagues [5]. Briefly, overnight NYB bacterial cultures were washed in sterile 0.9% NaCl solution and the resulting bacterial suspensions adjusted to OD_600_ of 0.1. Ten µL of the adjusted suspension (~10^6^ bacteria) were inoculated onto a pellet (diameter of 8 mm) of artificial diet [6]. The same volume of sterile NaCl solution was used as negative control. The cell densities of the bacterial inocula were assessed by dilution plating onto NA plates and counting of the colony forming units (CFU). Food pellets were placed onto sterile moisturised filter papers in six-well plates (Greiner bio-one, NC USA). Larvae were kept individually to avoid cannibalism. All the larvae were then placed in a Percival PGC-7L2 plant growth chamber (70% of humidity, 22 °C / 18 °C, 18h / 6 h light / dark photoperiod). Larval survival was assessed daily during six days by assessing their reaction to poking. Three independent feeding experiments were performed.

*Injection*

Fourth-instar *Pieris brassicae* larvae were used to evaluate the ability of the selected *Pseudomonas* strains to cause a systemic infection. Cells harvested from NYB cultures were washed using sterile 0.9% NaCl solution and adjusted to obtain bacterial suspensions of 2.5 · 10^4^ cells/mL. Approximatively 10^2^ cells contained in 2.5 µL suspension were injected at the level of the second proleg directly into the circulatory cavity (hemocoel) of the larvae [6]. Control larvae were injected with the same volume of sterile 0.9% NaCl. Serial dilutions of the inocula suspensions were plated onto NA plates for CFU counting as a control. Nine to 12 larvae were used per condition. Following injection, larvae were fed with a piece of pesticide-free cabbage and incubated as previously described. The survival of the larvae was monitored hourly, starting at 19 h post injection. Three independent injection experiments were performed.

*Colonisation*

Six third-instar larvae were starved for 18 h before being provided with a piece (Approx. 10 mg) of artificial diet (see above) inoculated with 5 µL of NaCl 0.9% containing ~10^7^ bacterial cells. Serial dilutions of the inocula suspensions were plated onto NA plates for CFU counting as a control. After 24 h following oral feeding, we selected larvae that had ingested all the artificial diet and dissected them to collect the haemolymph and the entire gut. Guts were placed into tubes containing 900 µl of 0.9% NaCl solution and glass beads, and were then homogenised using a Fast-Prep24 5G homogenizer (2 cycles of homogenization at 6 m/s for 40 s, MP Biomedicals). Gut and haemolymph samples were serially diluted and plated onto NA supplemented with 100 µg/mL of ampicillin and 30 µg/mL of chloramphenicol (specific selection of the different *Pseudomonas* strains) and incubated at 30°C. The experiment was repeated three times.

*Data analyses*

Larval survival data were represented using Kaplan-Meier plots and statistical differences were assessed using a log-rank test with fdr *p*value correction (survival v.3.3-1, survminer v0.4.9 and multicomp v1.4-20 R packages). To evaluate the differences between experiments, data was adjusted to a “proportional hazard survival regression” with the mixed-effect cox model using the different experiments as a random variable (coxme v.2.2-17 R package). The model showed that the probability that a larva died due to an effect of the experiment was 12.7% in injection and 11.6% in feeding experiments. Number of alive and dead larvae were counted at the midpoint between the first and the last death and compared using Pearson’s chi-square test (rstatix 0.7.0 R package). LT50 values were estimated using the ecotox v.1.4.4 R package with the LT_probit option [7]. Values were considered significantly different when there was no overlap between the 95% intervals of each strain. Treatments with no dead larvae were removed from the LT50 calculation.

**References**

1. Alouane T, Rimbert H, Fabre F, Cambon F, Langin T, Bonhomme L. Genome sequence of *Fusarium graminearum* strain MDC_Fg1, isolated from bread wheat grown in France. Microbiology Resource Announcements. 2018;7(19):e01260-18.

2. Maurhofer M, Keel C, Haas D, Défago G. Influence of plant species on disease suppression by *Pseudomonas fluorescens* strain CHA0 with enhanced antibiotic production. Plant Pathology. 1995;44(1):40-50.

3. Besset-Manzoni Y, Joly P, Brutel A, Gerin F, Soudiere O, Langin T, et al. Does *in vitro* selection of biocontrol agents guarantee success *in planta*? A study case of wheat protection against *Fusarium* seedling blight by soil bacteria. PLoS One. 2019;14(12):e0225655.

4. Sprouffske, Kathleen, et Andreas Wagner. « Growthcurver: an R package for obtaining interpretable metrics from microbial growth curves ». BMC Bioinformatics 2016; 17:1;172.

5. Flury P, Vesga P, Dominguez-Ferreras A, Tinguely C, Ullrich CI, Kleespies RG, et al. Persistence of root-colonizing *Pseudomonas protegens* in herbivorous insects throughout different developmental stages and dispersal to new host plants. The ISME Journal. 2019;13(4):860-72.

6. Vacheron J, Péchy-Tarr M, Brochet S, Heiman CM, Stojiljkovic M, Maurhofer M, et al. T6SS contributes to gut microbiome invasion and killing of an herbivorous pest insect by plant-beneficial *Pseudomonas protegens*. The ISME Journal. 2019;13(5):1318-29.

7. Hlina BL, Birceanu O, Robinson CS, Dhiyebi H, Wilkie MP. The relationship between thermal physiology and lampricide sensitivity in larval sea lamprey (*Petromyzon marinus*). Journal of Great Lakes Research. 2021;47:S272-S84.

**Supplementary Figure 1**

| 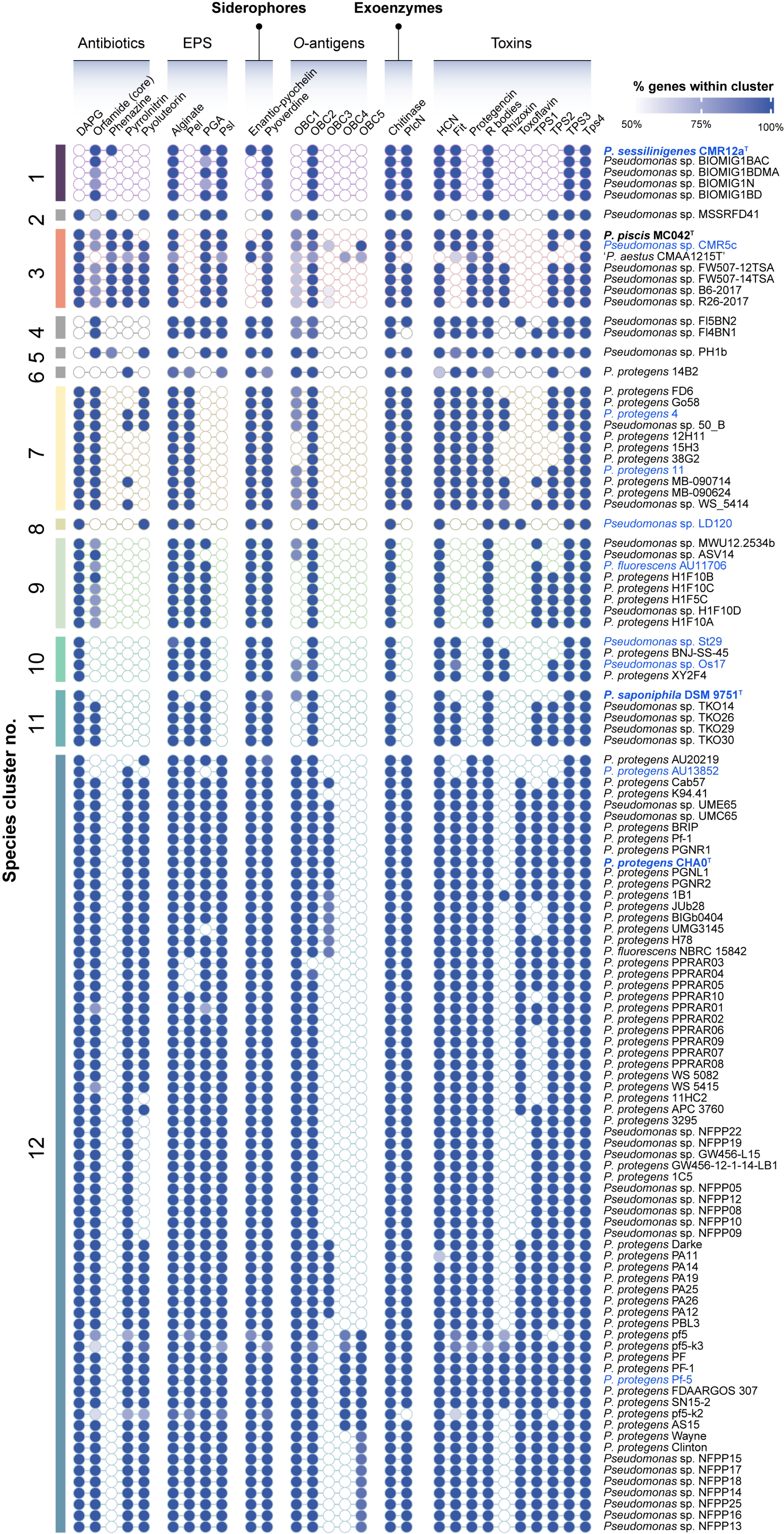 |
| --- |
| **Supplementary Figure 1** caption on next page. |
| **Supplementary Figure 1.** **Distribution of characters of interest in 115 *Pseudomonas protegens* SG genomes**. The scale shows the percentage of proteins within the cluster of proteins encoded in the strain genome. Only percentages ≥ 50% are considered. Strains phenotypically characterized in this study are highlighted in blue. DAPG (2,4-diacetylphloroglucinol): PhlABCDFGHI; HCN (hydrogen cyanide): HcnABC; orfamide: OfaABC+PPRCHA0_2164+PPRCHA0_2169-70; phenazine: PhzABCDEFGH; pyrrolnitrin: PrnABCD; pyoluteorin: PltADEGIJLMNORZ; alginate: AlgA1DEFGIKLQRUWXZ + Alg44; Pel (pellicle locus): PelABCDEFG; PGA (poly-N-acetyl-glucosamine): PgaABCD; Psl (polysaccharide synthesis locus): PslCDEFGHIJK; enantio-pyochelin: PchABCDFER; pyoverdine: PvdAEHJMOPQSTY; *O*-antigen biosynthetic cluster 1 (OBC1): PPRCHA0_2061-63 + PPRCHA0_2065-67; OBC2: PPRCHA0_3087 + PPRCHA0_3089-80 + PPRCHA0_3096 + PPRCHA0_3099 + PPRCHA0_3091-95 + PPRCHA0_3097-98 + PPRCHA0_3100-01; OBC3: AlgA3 + PPRCHA0_1950 + PPRCHA0_1954 + PPRCHA0_1957-59 + PPRCHA0_1962-64; OBC4: PFL_5482-86 + PFL_5488 + PFL_5490 + PFL_5492 + PFL_5496; OBC5: PFL_5092-93 + PFL_5097-5107; chitinase: ChiCD, Fit toxin: FitABCDEGH; protegenicin: PPRCHA0_0266-71; R bodies: RebB1B2 + PPRCHA0_0183-85; rhizoxin: RzxABCDEFGHI; toxoflavin: ToxABCDEGM; two-partner secretion pore-forming toxin (TPS) 1: TpsB1; TPS2: TpsA2B2; TPS3: TpsB3; TPS4: TpsA4B4. |

| 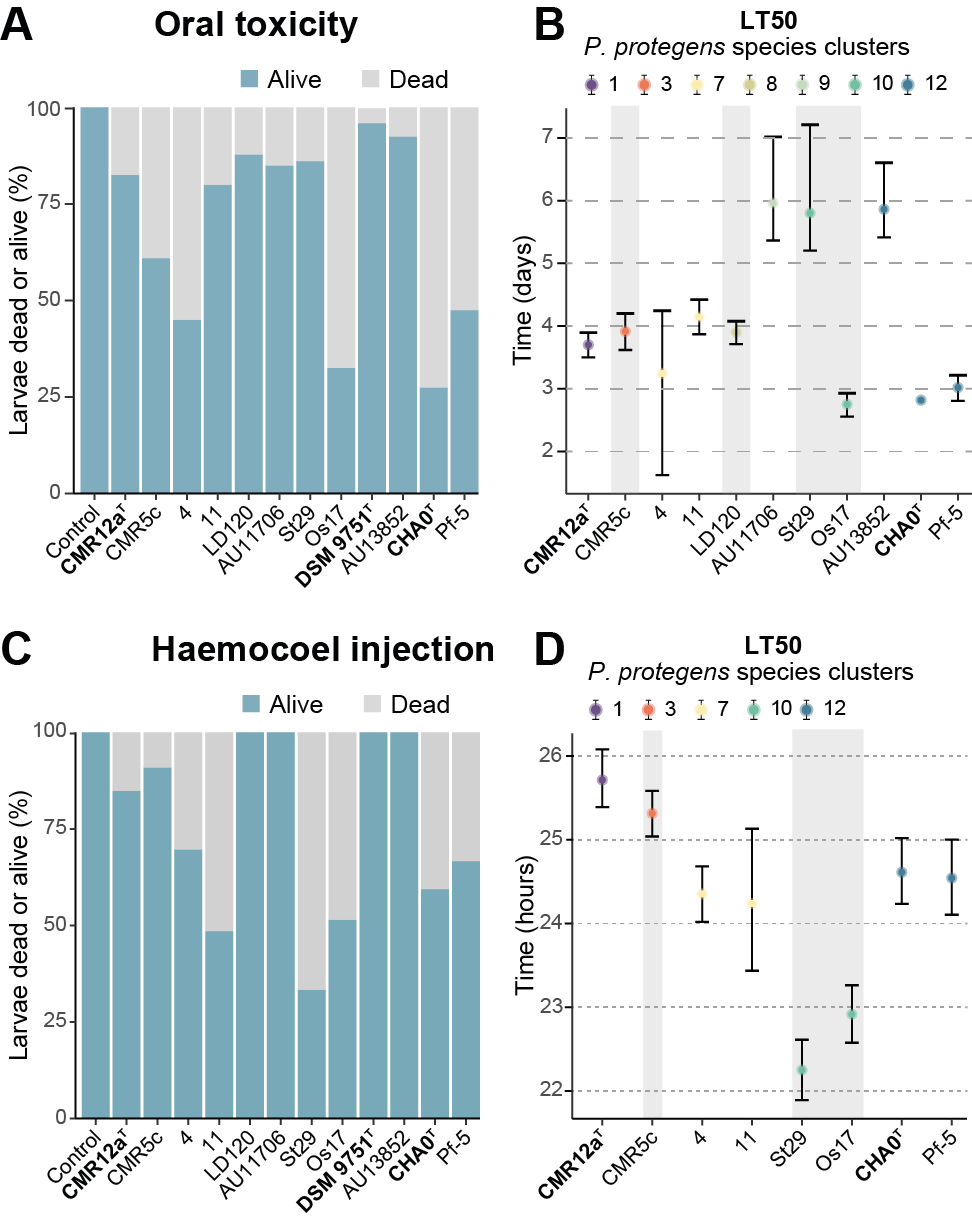 |
| --- |
| **Supplementary Figure 2.** **Mortality of *Pieris brassicae* larvae upon feeding or injection with 12 *Pseudomonas* strains**. (**A, C**) Percentage of alive (blue) or dead (grey) larvae counts at 3.5 days post oral administration or 23.75 h post injection, respectively. The respective time was chosen as the middle point between the first larval death and the end of the experiment. (**B, D**) Median lethal time (LT50) values of larvae for each *Pseudomonas* strain with 95% confidence intervals (CI). Treatments where no larvae died were removed from the LT50 analysis. LT50 values and confidence intervals are shown in the **Supplementary Table 10**. Strain names written in bold correspond to type strains. |

| 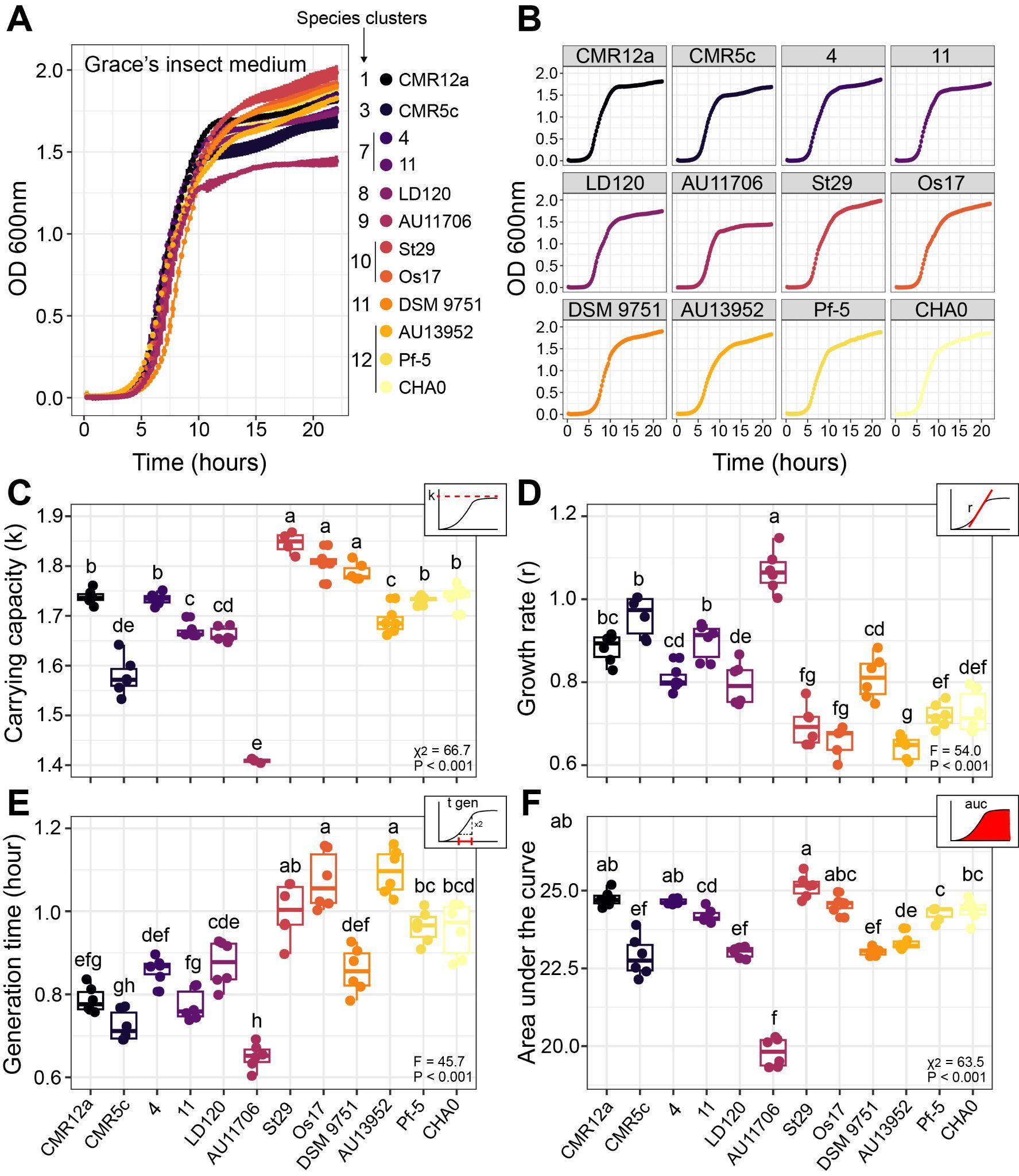  **Supplementary Figure 3.** **Growth parameters of the 12 *Pseudomonas* strains in Grace’s insect medium**. (**A**) Growth kinetic of the 12 *Pseudomonas* strains in Grace’s insect medium. (**B**) Individual visualisation of the growth kinetic in Grace’s insect medium. (**C-F**) Growth parameters extracted from the growth kinetics: (**C**) carrying capacity; (**D**) growth rate; (**E**) generation time; (**F**) area under the growth curve. Statistical differences between the strains were assessed by ANOVA coupled with Tukey’s HSD test or with Kruskal-Wallis test coupled with pairwise comparisons and are indicated with different letters. Two biological independent experiments were performed each with three technical replicates. |
| --- |

| 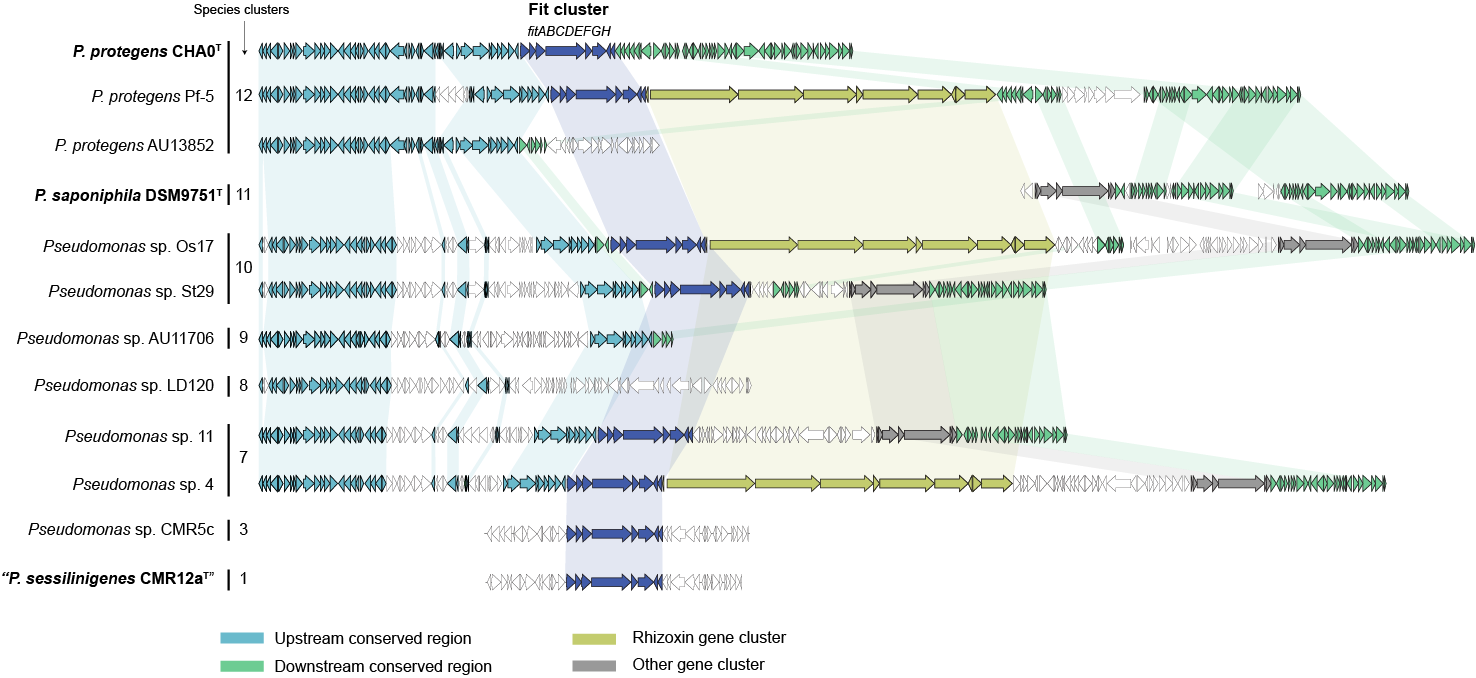 |
| --- |
| **Supplementary Figure 4.** Syntenic organisation of the Fit toxin gene cluster and flanking regions in the 12 *Pseudomonas* strains phenotypically characterised in this study. Strain names highlighted in bold correspond to type strains. |

**
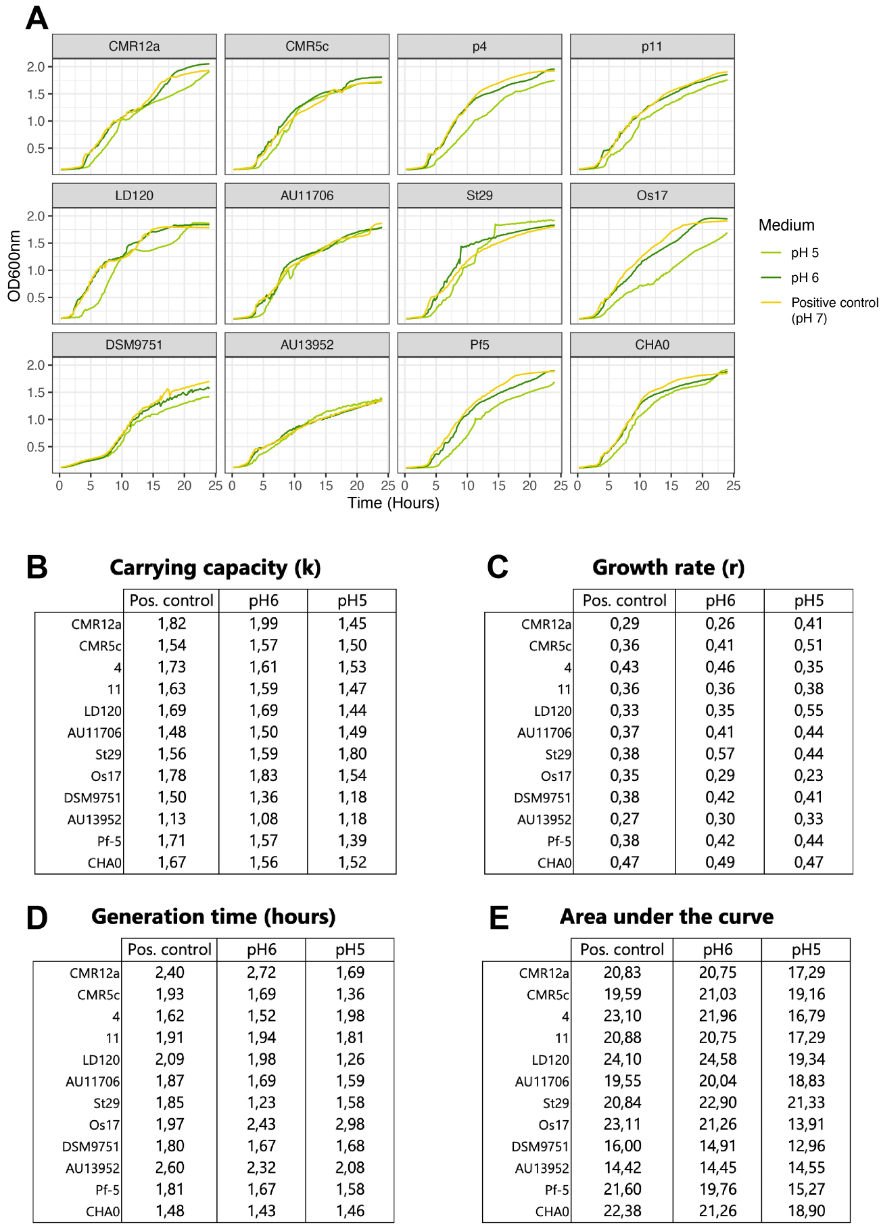
**

**Supplementary Figure 5.** **Growth parameters of the 12 selected *Pseudomonas* strains at different pH based on data extracted from the Biolog plates**. (**A**) Individual visualisation of the growth kinetic in the different pH media. (**B-E**) Growth parameters extracted from the growth kinetics: (**B**) carrying capacity; (**C**) growth rate; (**D**) generation time; (**E**) area under the growth curve.
